# Clinical Study on the Prevention of High-Risk Pulmonary Nodule Progression With Yifei Sanjie Pill: Protocol for a Multicenter Randomized Controlled Trial

**DOI:** 10.2196/78534

**Published:** 2026-07-15

**Authors:** Bo An, Jietao Lin, Jingrui Wang, Zhiqiang Chen, Minyi Guan, Yanlong Li, Xiangjun Qi, Lingling Sun, Lizhu Lin

**Affiliations:** 1 The First Affiliated Hospital of Guangzhou University of Chinese Medicine, Guangdong Clinical Research Academy of Chinese Medicine, No. 16 Airport Road, Baiyun District, Guangzhou, 510400, China, 86 15625210828 ext 15625210828; 2 Department of Oncology, The First Affiliated Hospital of Guangzhou University of Chinese Medicine, Guangzhou, China; 3 Guangzhou University of Chinese Medicine, Guangzhou, China

**Keywords:** Yifei Sanjie Pill, clinical study, protocol, pulmonary nodule, traditional Chinese medicine

## Abstract

**Background:**

High-risk subsolid pulmonary nodules, especially mixed ground-glass nodules, can represent precancerous or early-stage lung adenocarcinoma spectrum lesions. Standard management relies mainly on risk stratification, repeated thin-slice computed tomography, and invasive diagnosis or surgery when progression suggests malignancy. Safe adjunctive pharmacologic options to reduce nodule progression remain limited. Yifei Sanjie Pill (YFSJ) is an 8-herb traditional Chinese medicine formula that has been previously studied as an adjunctive treatment for non–small cell lung cancer. However, its effect on high-risk pulmonary nodules—a distinct clinical condition—has not yet been investigated.

**Objective:**

This protocol describes a multicenter randomized trial evaluating the efficacy, safety, and exploratory mechanisms of YFSJ for preventing high-risk pulmonary nodule progression.

**Methods:**

Adults aged 18 to 80 years with single or multiple mixed ground-glass nodules measuring ≤8 mm will be randomized in a 1:1 ratio to receive YFSJ optimized formula granules or matched placebo granules, 2 packets twice daily for 6 months. Both groups will receive guideline-based computed tomography surveillance and any clinically indicated standard diagnostic or therapeutic care. The primary outcome is the 2-year pulmonary nodule progression rate, defined as an increase in the lesion’s longest diameter of ≥2 mm, an increase in the solid component of ≥1 mm, or the appearance of a new solid component. Secondary outcomes include lung cancer detection rate, surgery rate, 6-month nodule size and volume change, artificial intelligence–based malignancy risk index, traditional Chinese medicine symptom score, Hospital Anxiety and Depression Scale (HADS) score, Pittsburgh Sleep Quality Index (PSQI) score, safety indicators, and exploratory plasma biomarkers. The primary analysis will use the full analysis set, Kaplan-Meier estimates, log-rank testing, and Cox regression adjusted for center and key covariates; repeated outcomes will be analyzed using mixed-effects models.

**Results:**

The study was funded in 2022 and approved by the Research Ethics Committee of the First Affiliated Hospital of Guangzhou University of Chinese Medicine (K-2022‐131-XZ-01). Recruitment started in June 2023 and was scheduled for completion in December 2025. As of April 2026, a total of 589 participants have completed randomization. The final 2-year follow-up is scheduled for December 2027. Database lock and primary analysis are planned for early 2028, and submission of the first results is expected in spring or summer 2028.

**Conclusions:**

This trial will determine whether adding YFSJ to guideline-based surveillance can reduce high-risk pulmonary nodule progression and provide mechanistic evidence for integrative early lung cancer prevention.

## Introduction

Lung cancer remains a major cancer burden in China. National mortality surveillance data showed that cancer-related deaths in China reached 2,397,772 in 2020, a 21.6% increase compared with 2005, and that tracheal, bronchial, and lung cancers remained among the leading causes of cancer mortality [1]. Because prognosis depends strongly on stage, strategies that identify and manage early lung cancer spectrum lesions are essential.

The broader use of low-dose computed tomography (CT) has increased the detection of pulmonary nodules, including pure ground-glass nodules and mixed ground-glass nodules [2-4]. Most small nodules are benign or indolent, but persistent subsolid nodules may correspond to atypical adenomatous hyperplasia, adenocarcinoma in situ, minimally invasive adenocarcinoma, or invasive adenocarcinoma. The National Comprehensive Cancer Network (NCCN) and Fleischner Society lung nodule surveillance guidelines emphasize risk-adapted surveillance, avoidance of unnecessary invasive procedures, and timely intervention when growth or the development of a solid component increases the likelihood of malignancy [5,6].

The target population in this study is adults with high-risk pulmonary nodules rather than patients with confirmed advanced lung cancer. For nodules ≤8 mm, immediate resection may overtreat indolent disease, whereas surveillance alone does not actively address biological progression. Long-term follow-up studies also indicate that some ground-glass nodules may grow after prolonged stability, supporting the need to investigate safe interventions that can be used alongside guideline-based CT surveillance [7].

Artificial intelligence (AI)–assisted CT analysis is increasingly used to quantify pulmonary nodule size, volume, density, morphology, and malignancy probability. Recent studies have shown that AI and data-driven risk stratification can support risk classification and may outperform size-only approaches in selected settings [8,9]. In this trial, AI-derived nodule volume and malignancy risk index are included as secondary imaging outcomes to provide quantitative and reproducible assessments in addition to conventional radiologist interpretation.

Psychological distress is not the primary target of this trial, but it is clinically relevant for patients who undergo repeated surveillance for indeterminate pulmonary nodules. Previous studies have reported anxiety, depression, sleep disturbance, and reduced quality of life in patients with pulmonary nodules [10,11]. Therefore, the Hospital Anxiety and Depression Scale (HADS) and Pittsburgh Sleep Quality Index (PSQI) are included as secondary patient-reported outcomes, whereas the primary end point remains objective nodule progression.

Traditional Chinese medicine (TCM) is widely used in China as part of integrative oncology care. Network pharmacology and experimental studies suggest that TCM formulas may influence multiple pathways involved in pulmonary nodule and early lung cancer biology, including inflammation, cell proliferation, apoptosis, immune regulation, and tumor microenvironment remodeling [12,13]. These multitarget effects provide a biological rationale for evaluating TCM as an adjunct to surveillance in high-risk pulmonary nodules, provided that rigorous randomized evidence is generated.

Yifei Sanjie Pill (YFSJ), also referred to as the Yi-Fei-San-Jie formula, is an 8-herb formula developed from the clinical experience of Professor Lizhu Lin. Prior studies have evaluated YFSJ as an adjunctive treatment for non–small cell lung cancer [14-16], but this study investigates a different clinical population: patients with high-risk pulmonary nodules. The source formula contains *Sarcandra glabra*, Bombyx batryticatus, *Ranunculus ternatus*, *Iphigenia indica*, Bulbus *Fritillariae thunbergii*, *Pinellia ternata*, *Ganoderma lucidum*, and *Panax quinquefolius* [17]. Recent mechanistic works have identified bioactive components, such as ginsenosides, peimisine, and peimine, and have suggested regulation of transforming growth factor-β signaling, tumor immune escape, deoxycholic acid metabolism, and the TGR5/STAT3/PD-L1 (Takeda G protein-coupled receptor 5, signal transducer and activator of transcription 3, and programmed death-ligand 1) axis [17,18]. These findings support the plausibility of YFSJ in modulating early lung cancer–related biology, but they do not establish efficacy in preventing pulmonary nodule progression.

The purpose of this multicenter, randomized, double-blind, and placebo-controlled trial is to evaluate whether YFSJ, administered for 6 months in addition to guideline-based surveillance, reduces the 2-year progression rate of high-risk pulmonary nodules compared with placebo plus the same surveillance strategy. We hypothesize that YFSJ will reduce nodule progression without increasing clinically meaningful adverse events and will improve selected TCM symptoms, psychological distress, sleep quality, and exploratory plasma biomarkers related to phlegm-heat-toxin pathogenesis.

## Methods

### Study Governance

The research team for this study includes experts from institutions such as Guangzhou University of Chinese Medicine and Nanjing University of Chinese Medicine, with defined roles such as principal investigator and study coordinators responsible for data collection, patient management, and analysis. The trial sponsor, Guangzhou University of Chinese Medicine First Affiliated Hospital, oversees the design, management, data analysis, and interpretation of the trial, including the publication of results. The coordinating center for the trial, also located at Guangzhou University of Chinese Medicine First Affiliated Hospital, ensures the trial’s overall operation, managing data collection, patient follow-up, and protocol compliance in collaboration with various teams. Contact information for the principal investigators and coordinators is provided to facilitate clear communication. This study protocol was developed in accordance with the SPIRIT (Standard Protocol Items: Recommendations for Interventional Trials) 2013 Statement. The completed SPIRIT checklist is provided in Checklist 1.

### Ethical Considerations

The clinical trial protocol (version 3.0, March 20, 2023) was approved by the Research Ethics Committee of the First Affiliated Hospital of Guangzhou University of Chinese Medicine (approval number: K-2022‐131-XZ-01). The trial is registered in the Chinese Clinical Trial Registry (ChiCTR2300069570) and will be conducted in accordance with the Declaration of Helsinki and Good Clinical Practice principles.

Written informed consent will be obtained before any trial-specific procedures. Investigators will explain the study’s purpose, interventions, expected benefits, foreseeable risks, alternative management options, confidentiality protections, voluntary participation, and the right to withdraw without affecting usual medical care. Additional consent will be obtained for the storage and exploratory analysis of plasma samples.

Participant data will be entered into an electronic data capture (EDC) system using coded study identifiers. Direct identifiers will be stored separately from research data, access will be restricted to authorized study personnel, and analyses will use deidentified datasets. Biological samples will be labeled with coded identifiers and used only for approved analyses described in the protocol or subsequent ethics-approved amendments.

Participants will not be denied clinically indicated diagnostic evaluations or standard treatments. Study medication and protocol-specified assessments will be provided by the study. No direct financial incentives are planned. Medical management and compensation for trial-related injuries will be provided according to institutional and national regulations.

### Case Selection

#### Sample Size Estimation

**Figure 1.**
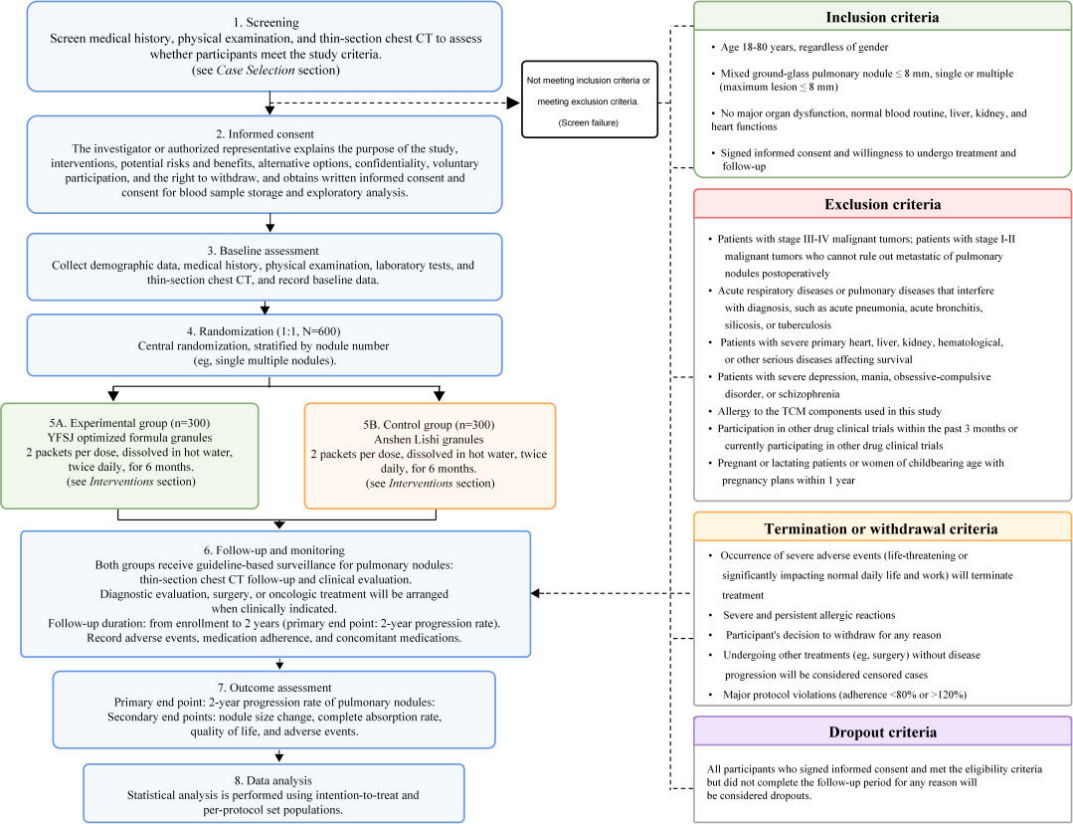
Clinical study flowchart. CT: computed tomography; TCM: traditional Chinese medicine; YFSJ: Yifei Sanjie Pill.

This trial uses the 2-year disease progression rate of pulmonary nodules as the primary end point. Previous studies have reported a 2-year progression rate of ≤8 mm mixed ground-glass nodules at approximately 24% [19]. Assuming a 2-year progression rate of 14% in the intervention group, with 80% power, a 5% false-positive error rate (2-sided), and a dropout rate controlled within 20%, a total of 596 participants need to be enrolled. The trial will include 600 patients, with 300 in the experimental group and 300 in the control group (Figure 1).

#### Inclusion Criteria

Participants will be eligible for enrollment in the study if they meet all of the following inclusion criteria:

- Age 18 to 80 years, regardless of gender
- ≤8 mm mixed ground-glass pulmonary nodule, single or multiple (with the largest lesion ≤8 mm)
- No major organ dysfunction, normal blood routine, liver, kidney, and heart functions
- Signed informed consent and willingness to undergo treatment and follow-up

#### Exclusion Criteria

Participants meeting any of the following criteria will be excluded from the study:

- Patients with stage III-IV malignant tumors or patients with stage I-II malignant tumors who cannot rule out metastasis of pulmonary nodules postoperatively
- Acute respiratory diseases or pulmonary diseases that interfere with diagnosis, such as acute pneumonia, acute bronchitis, silicosis, and tuberculosis
- Patients with severe primary heart, liver, kidney, hematological, or other serious diseases affecting survival
- Patients with clinically significant immune disease, autoimmune disease, immunodeficiency, or current systemic immunosuppressive or immunomodulatory treatment that could affect safety assessments or immune-related outcomes
- Patients with severe depression, mania, obsessive-compulsive disorder, or schizophrenia
- Allergy to the TCM components used in this study
- Patients who have participated in other drug clinical trials in the past 3 months or are currently participating in other drug clinical trials
- Pregnant or lactating patients, as well as women of childbearing age with pregnancy plans within 1 year

#### Termination or Withdrawal Criteria

Participants will be withdrawn from the study or have their treatment discontinued if any of the following conditions occur:

- Patients experiencing severe adverse events (life-threatening or significantly impacting normal daily work and life) will discontinue treatment
- Patients experiencing severe and persistent allergic reactions will cease treatment and be considered ineffective cases
- Patients unwilling or unable to continue clinical trials for any reason and requesting withdrawal
- Patients who undergo other treatments, such as surgery, without disease progression will be considered censored cases
- Significant protocol violations (adherence <80% or >120%)

#### Dropout Criteria

All participants who signed the informed consent and were eligible for observation but did not complete the observation period for any reason will be considered dropouts.

### Interventions

#### Guideline-Based Surveillance

Both groups will receive guideline-based surveillance for pulmonary nodules, including thin-slice chest CT follow-up, clinical evaluation, and referral for diagnostic work-up, surgery, or oncologic treatment when clinically indicated. The study intervention is not a substitute for standard care. If lung cancer is suspected or confirmed during follow-up, the participant will be managed according to contemporary clinical guidelines and local multidisciplinary recommendations; such events will be recorded as trial outcomes.

#### YFSJ Optimized Formula Granules

Participants allocated to the experimental group will take optimized YFSJ formula granules—2 packets per dose, dissolved in hot water—twice daily for 6 months. The source formula is composed of 8 herbs as described in Table 1; the crude-drug equivalent dose and the final granule extraction ratio or packet weight should be verified against the investigational product specification.

**Table 1.** Composition of the Yifei Sanjie Pill (YFSJ) source formula^a^.

Number	Herbal component	Latin or pharmaceutical name	Crude-drug equivalent in source formula, g
1	Sarcandra glabra	*Sarcandra glabra* (Thunb.) Nakai	15
2	Bombyx batryticatus	Bombyx batryticatus	10
3	*Ranunculus ternatus*	*Ranunculus ternatus* Thunb.	30
4	*Iphigenia indica*	*Iphigenia indica* Kunth	15
5	Fritillaria bulb	Bulbus *Fritillariae thunbergii*	15
6	*Pinellia ternata*	*Pinellia ternata* (Thunb.) Breit.	15
7	*Ganoderma lucidum*	*Ganoderma lucidum* (Leyss. ex Fr.) Karst.	30
8	American ginseng	*Panax quinquefolius* L.	15

a The investigational granules are manufactured by Jiangyin Tianjiang Pharmaceutical Co, Ltd. The batch number, quality-control fingerprint, heavy metal or pesticide testing, granule extraction ratio, and gram weight per packet will be recorded in the trial master file.

#### Placebo Granules

Participants allocated to the control group will take matched placebo granules—2 packets per dose, dissolved in hot water—twice daily for 6 months. The placebo contains 5% of the active YFSJ optimized granules, along with flavoring agents, starch, and colorants to match the appearance, taste, smell, packaging, and administration schedule as closely as possible. The placebo is used only in addition to guideline-based surveillance and does not withhold clinically indicated care.

#### Concomitant Medication Rules

All concomitant medications and treatments during the trial period (screening and treatment period) must follow the following rules:

1. In addition to the study medication, doctors should require patients to bring all medications they are taking to follow-up visits for checking. Essential medications or other treatments for concomitant diseases should be recorded in the case report form (CRF), including the medication name (or other treatment), dosage, frequency, and timing for analysis and reporting.
2. Patients may receive necessary treatment for other diseases or symptoms (unrelated to this condition), but the treatment must be truthfully and thoroughly recorded in the CRF.
3. During the trial, the use of other TCM prescriptions with known antitumor activity or with primary functions such as fever reduction (antipyretic), phlegm clearance (mucolytic), and lung function support (including bronchodilatory effects) is prohibited.

#### Randomization, Allocation Concealment, and Blinding

The random allocation sequence will be generated by an independent third-party statistician using a computer-based random number generator. Participants will be randomized in a 1:1 ratio to the YFSJ or placebo group. Allocation concealment will be maintained using sequentially numbered, sealed, and opaque envelopes or an equivalent centralized allocation procedure; the sequence will not be available to investigators responsible for the enrollment or outcome assessment.

The trial uses a double-blind design. Participants, treating clinicians, investigators, outcome assessors, data managers, and statisticians will remain blinded to group assignment until database lock, except in medical emergencies requiring unblinding. Study medications will be identical in packaging and labeled by coded box numbers. Emergency unblinding procedures will be documented, and the reason, date, and person performing the unblinding will be recorded.

### Outcome Measures and Rationale

#### Clinical Efficacy Outcomes

The outcomes were selected to evaluate YFSJ across 4 levels: (1) lesion-level clinical efficacy, using objective nodule progression as the primary end point; (2) imaging-based quantitative change, including CT and AI-derived size, volume, and malignancy risk; (3) patient-reported symptoms, psychological distress, and sleep quality, reflecting the burden of long-term surveillance; and (4) exploratory biological mechanisms that may link the TCM phlegm-heat-toxin pattern with measurable inflammatory, immune, metabolic, and endoplasmic-reticulum (ER) stress pathways. This hierarchy ensures that the trial hypothesis remains centered on disease progression while allowing secondary and mechanistic analyses.

#### Primary End Point

The primary outcome is the 2-year lung nodule progression rate. Progression of a ground-glass nodule is defined as an increase in the longest diameter of the lesion by ≥2 mm, an increase in the solid component by ≥1 mm, or the appearance of a new solid component. When multiple nodules are present, the largest or clinically dominant nodule will be used for primary assessment, and changes in other nodules will be documented as supportive information.

#### Secondary End Points

The following secondary end points will be assessed:

1. Lung cancer detection rate during the 2-year follow-up, confirmed by histopathology when available or by multidisciplinary clinical diagnosis when pathology is unavailable
2. Surgery rate and reason for surgery, including progression, patient preference, suspected malignancy, or other clinical indications
3. The 6-month change in nodule size, solid-component size, CT density, and AI-derived nodule volume
4. AI malignancy risk index, defined as the algorithm-generated malignancy probability or risk score for the target nodule. The score scale will be reported according to the software output, typically 0 to 100 or a calibrated probability, with higher values indicating higher predicted malignancy risk. The AI system is an independently developed deep learning–based automatic lung nodule segmentation technology (patent application number: 202510952658.0)
5. Pulmonary nodule symptom scale, based on the trial-specific TCM symptom scale for cough, sputum, chest tightness, fatigue, dry mouth or throat, heat signs, and other symptoms related to the phlegm-heat-toxin pattern. This scale was developed using the Delphi expert consensus method to provide a standardized symptomatic evaluation tool for pulmonary nodule patients treated with TCM or integrated traditional Chinese and Western medicine interventions. Each item, scoring range, and minimal clinically important difference will be listed in the final protocol appendix [20]
6. HADS, a 14-item instrument with anxiety and depression subscales [21]
7. PSQI, a validated self-report measure of sleep quality [22]

#### Safety Outcomes

The following safety outcomes will be assessed throughout the study:

1. Vital signs, including pulse, respiratory rate, blood pressure, and temperature
2. Blood routine, liver function, kidney function, urinalysis, stool examination, and electrocardiogram ()
3. All adverse events and serious adverse events, including onset date, duration, severity, relationship to study medication, action taken, outcome, and whether unblinding was required

### AI-Assisted CT Assessment

Chest CT will be performed using a thin-slice protocol harmonized across participating centers as far as feasible. CT acquisition parameters, reconstruction kernel, slice thickness, and scanner model will be recorded. Radiologists, blinded to treatment allocation, will assess nodule type, longest diameter, solid-component size, morphology, and progression status.

The AI-assisted nodule analysis system, an independently developed deep learning–based automatic lung nodule segmentation technology (patent application number: 202510952658.0), will segment the target nodule and extract quantitative features, including nodule volume, longest diameter, solid-component size, density, and malignancy risk index. The AI output will not replace clinical judgment or standard reporting. Discrepancies between radiologist assessment and AI output will be reviewed by a blinded adjudication committee or a senior radiologist according to a prespecified adjudication procedure.

### Follow-Up and Data Collection

Baseline data will include demographics, smoking history, comorbidities, prior cancer history, medication history, physical examination, vital signs, blood routine, liver function, kidney function, urinalysis, stool examination, tumor markers, ECG, chest CT, AI-derived nodule variables, TCM symptom score, HADS, and PSQI. Follow-up visits will occur at 3 and 6 months during the intervention period and every 6 months thereafter until nodule progression, surgery, withdrawal, death, loss to follow-up, or study completion. The trial protocol is version 3.0 (March 20, 2023), and the schedule of study procedures is shown in Table 2.

**Table 2.** Schedule of study procedures, assessments, and follow-up visits.

Procedure	Screening/baseline	Randomization	Month 3	Month 6	Month 12	Month 18	Month 24/ end point
Eligibility	✓						
Consent	✓						
Demog.^a^	✓						
Random.^b^		✓					
Study drug		Start	Continue	End			
Meds^c^	✓	✓	✓	✓	✓	✓	✓
Vitals or AEs^d^	✓		✓	✓	✓	✓	✓
Labs or ECG^e^	✓		✓	✓	As indicated	As indicated	✓
CT^f^	✓			✓	✓	✓	✓
AI^g^	✓			✓	✓	✓	✓
TCM^h^ symptoms	✓		✓	✓	✓	✓	✓
HADS^i^ or PSQI^j^	✓		✓	✓	✓	✓	✓
Plasma	✓			✓			Optional or if specified
End point							✓

a Demog.: demographics and medical history.

b Random.: randomization and dispensing.

c Meds: concomitant medication review.

d AEs: adverse events.

e ECG: electrocardiogram.

f CT: computed tomography.

g AI: artificial intelligence.

h TCM: traditional Chinese medicine.

i HADS: Hospital Anxiety and Depression Scale.

j PSQI: Pittsburgh Sleep Quality Index.

### Plasma Collection and Mechanistic Analysis

Plasma samples will be collected at baseline and after 6 months of medication. A 10-mL peripheral blood sample will be collected in ethylenediaminetetraacetic acid tubes, centrifuged at 1500*g* for 10 minutes, aliquoted, and stored at −80 °C until analysis. The phrase “phlegm-heat-toxin pathogenesis” refers to a TCM theoretical pattern characterized clinically by phlegm accumulation, heat-related symptoms, toxin-like pathogenic stagnation, and impaired qi regulation. In this trial, the concept will be operationalized using TCM symptom scores and exploratory plasma markers rather than treated as a directly observable biomedical entity. The exploratory biological analysis will evaluate whether YFSJ changes biomarker patterns associated with inflammatory activation, immune dysregulation, oxidative stress, ER stress, and bile-acid metabolism. Candidate assays include multiplex or enzyme-linked immunosorbent assay measurement of interleukin-6 (IL-6), IL-1-β, tumor necrosis factor-α, IL-8, interferon-γ, C-reactive protein, vascular endothelial growth factor, and other prespecified cytokines; targeted liquid chromatography-mass spectrometry or mass spectrometry analysis of bile-acid metabolites such as deoxycholic acid, taurochenodeoxycholic acid, and glycocholic acid; and validated assays for ER stress–related markers such as glucose-regulated protein 78 or binding immunoglobulin protein, CCAAT/enhancer-binding homologous protein, activating transcription factor 6, spliced X-box-binding protein 1, and related pathway proteins when measurable in plasma or extracellular vesicles. Untargeted metabolomics or proteomics may be used to generate hypotheses, with false discovery rate control for multiple testing.

### Adherence and Retention Strategies

Participants will receive phone reminders before scheduled visits. Participants who miss a visit will be contacted by phone, text message, or home visit, as appropriate, to determine the reason and encourage continued participation. Medication adherence will be assessed through returned packet counts and participant report. Available imaging, laboratory, and questionnaire data will be collected at withdrawal whenever consent permits.

### Data Management and Monitoring

Data will be recorded in standardized CRFs and entered into an EDC system. Research staff will receive uniform training in eligibility confirmation, informed consent, CT data collection, AI output recording, questionnaire administration, adverse event reporting, and data entry. Data queries will be resolved before the database lock. The sponsor and centralized clinical management team will perform regular internal monitoring to ensure participant safety, protocol adherence, and data quality.

Because the intervention is noninvasive and the risk is considered low, an independent data monitoring committee is not planned. Serious adverse events will be reported to the ethics committee and sponsor according to the protocol and regulatory timelines. Emergency unblinding will be allowed only when knowledge of the treatment assignment is essential for clinical management.

### Statistical Analysis

Data will be collected at baseline and during follow-up visits using standardized CRFs. Key data include demographics, clinical history, imaging findings (chest CT and AI-assessed nodule features), laboratory results (tumor markers, blood, urine, stool, liver and kidney function, and ECG tests), and patient-reported outcomes (TCM symptom scale, provided in Multimedia Appendix 1, HADS, and PSQI). To ensure data quality, assessors will receive uniform training, and duplicate measurements will be conducted where appropriate. All instruments used have been validated in prior clinical practice. Data collection forms are included in the protocol and managed through an EDC system.

Based on the actual number of participants enrolled in the 2 groups, dropout and exclusion cases, as well as demographic and other baseline characteristics, corresponding efficacy and safety analyses will be conducted. Descriptive statistical analysis will be used, with qualitative indicators described as frequency tables, percentages, or proportions; quantitative indicators will be described as mean (SD), median (IQR), and minimum and maximum values.

For primary end points and clinical efficacy analysis, the main and sensitivity analyses will be performed according to the full analysis set and per-protocol set, respectively. Safety analysis will be conducted using the safety set. The main analysis of the primary end point will use the log-rank test to compare the 2-year progression rates between groups, with Cox regression adjusting for covariates (eg, center) as a sensitivity analysis. Subgroup analyses will be conducted based on the center and whether >8 mm nodules were surgically removed. The malignancy risk index, quality-of-life scores at different time points, and other multiple measurement indicators will be analyzed using mixed-effects models. Eligibility-related protocol deviations, including any deviations from the retained immune disease exclusion criterion, will be documented. Participants with major eligibility deviations will be handled according to the prespecified analysis-set definitions, with sensitivity analysis based on the per-protocol set. Safety end points will summarize adverse reactions and events occurring during the study and compare rates between groups. Hypothesis testing will uniformly use 2-sided tests, providing test statistics and corresponding *P* values, with *P*<.05 considered statistically significant.

## Results

The study was funded in 2022 and approved by the Research Ethics Committee of the First Affiliated Hospital of Guangzhou University of Chinese Medicine (K-2022‐131-XZ-01). Grant support has been provided by the Guangdong Basic and Applied Basic Research Foundation (2022B1515230003), the Guangdong Special Support Program for Leading Talents (0720240115), the “China Joint Graduate School of Traditional Chinese Medicine” Science and Education Special Project (CI2023C030LH), and the Guangzhou Regional Clinical Major Innovation Technology Construction Project for Collaborative Traditional Chinese and Western Medicine (Joint Construction Project). Recruitment began in June 2023 and was scheduled for completion in December 2025. As of April 2026, 589 cases have been enrolled. The target sample size remains 600 participants.

Because the primary outcome is assessed over 2 years, the last primary end point visit is scheduled for December 2027 if the final participant was randomized in December 2025. Data cleaning and database lock are planned for early 2028, followed by the primary statistical analysis and submission of the first results manuscript in spring or summer 2028. No comparative efficacy results are reported in this protocol manuscript before the completion of follow-up and database lock.

## Discussion

### Principal Findings

This trial is designed to test the hypothesis that YFSJ, when added to guideline-based surveillance, can reduce the 2-year progression rate of high-risk pulmonary nodules compared with placebo combined with the same surveillance strategy. If the intervention reduces progression without increasing adverse events, YFSJ may provide a noninvasive adjunctive option for patients with small, high-risk, and mixed ground-glass nodules who do not yet meet the criteria for invasive treatment.

The primary end point is objective radiologic progression. Patient-reported psychological and sleep outcomes are included because prolonged surveillance may create distress, and symptom improvement is relevant to integrative care; however, these outcomes will be interpreted as secondary and supportive.

Existing pulmonary nodule guidance focuses on risk stratification, CT follow-up, and surgical or diagnostic intervention when features suggest malignancy [4-6]. Recent AI-based studies further show that integrating quantitative imaging, clinical features, and longitudinal data can refine malignancy risk classification [8,9]. However, these approaches mainly improve detection and decision-making; they do not provide pharmacological prevention of nodule progression.

YFSJ has been studied in non–small cell lung cancer models and adjunctive treatment settings, with evidence suggesting regulation of apoptosis, autophagy, and inflammatory pathways [14-18]. While those studies focused on established lung cancer, this trial extends this prior work to a prevention-oriented population with high-risk pulmonary nodules, using a randomized, double-blind, and placebo-controlled design to reduce bias.

Key strengths of this study include the multicenter, randomized, double-blind design; matched placebo control; objective 2-year radiologic primary end point; standard-of-care surveillance in both groups; AI-assisted quantitative imaging; patient-reported outcomes; and planned exploratory plasma analyses. The trial also explicitly distinguishes clinical efficacy end points from mechanistic hypotheses, which improves interpretability.

Several limitations should be considered. First, a 2-year follow-up may not capture all late-growing ground-glass nodules, which can progress after longer periods of stability. Second, AI-derived malignancy indices depend on the software, scanner protocol, and validation population; therefore, AI outputs will be interpreted as secondary quantitative measures rather than definitive diagnoses. Third, the placebo contains 5% active YFSJ ingredients to support blinding, which could attenuate between-group differences if low-dose exposure has biological activity. Fourth, the mechanistic biomarker analyses are exploratory and require confirmation in independent studies. Finally, the findings may be most generalizable to Chinese clinical settings, where TCM use and CT surveillance workflows are similar to those in participating centers.

The final results will be disseminated through peer-reviewed publications, conference presentations, trial registry updates, and communication with participating centers and relevant stakeholders. The full protocol, statistical analysis plan, deidentified dataset, and statistical code will be made available after the completion of the trial when permitted by ethics approval, consent, and applicable data governance rules.

### Conclusions

This protocol describes a multicenter randomized trial evaluating YFSJ for the prevention of high-risk pulmonary nodule progression. By combining guideline-based surveillance, objective CT progression end points, AI-assisted imaging, patient-reported outcomes, safety monitoring, and exploratory plasma biomarkers, this study will provide evidence on whether YFSJ is a safe and effective adjunctive strategy for patients with high-risk pulmonary nodules.

## Supplementary material

10.2196/78534 Multimedia Appendix 1 Pulmonary nodule traditional Chinese medicine symptom scale.

10.2196/78534 Checklist 1 SPIRIT checklist.

## Abbreviations

AI: artificial intelligence
CRF: case report form
CT: computed tomography
ECG: electrocardiogram
ER: endoplasmic reticulum
HADS: Hospital Anxiety and Depression Scale
IL-6: interleukin-6
NCCN: National Comprehensive Cancer Network
PSQI: Pittsburgh Sleep Quality Index
SPIRIT: Standard Protocol Items: Recommendations for Interventional Trials
TCM: traditional Chinese medicine
TGR5/STAT3/PD-L1: Takeda G protein-coupled receptor 5, signal transducer and activator of transcription 3, and programmed death-ligand 1
YFSJ: Yifei Sanjie Pill
