# Supplementary material for: Clinical Study on the Prevention of High-Risk Pulmonary Nodule Progression With Yifei Sanjie Pill: Protocol for a Multicenter Randomized Controlled Trial
Source: JMIR Res Protoc. 2026 Jul 15;15:e78534. doi: 10.2196/78534 (PMC13372260; doi:10.2196/78534)
Supplement: Multimedia Appendix 1 [file resprot-v15-e78534-s001.docx]

# Pulmonary Nodule Patient TCM Symptom Scale

## Part 1: How severe are your symptoms? (To be completed by the patient)

We would like to know the severity of the following symptoms over the past week. Please circle a number between 0 (no symptoms) and 10 (most severe imaginable) to indicate the severity of each symptom.

| Symptom | None |  |  |  |  |  |  |  |  |  | Most Severe |
| --- | --- | --- | --- | --- | --- | --- | --- | --- | --- | --- | --- |
| How severe was your cough? | 0 | 1 | 2 | 3 | 4 | 5 | 6 | 7 | 8 | 9 |  |
| How severe was your sputum production? | 0 | 1 | 2 | 3 | 4 | 5 | 6 | 7 | 8 | 9 |  |
| How severe was your chest tightness? | 0 | 1 | 2 | 3 | 4 | 5 | 6 | 7 | 8 | 9 |  |
| How severe was your chest pain? | 0 | 1 | 2 | 3 | 4 | 5 | 6 | 7 | 8 | 9 |  |
| How severe was your fatigue or weakness? | 0 | 1 | 2 | 3 | 4 | 5 | 6 | 7 | 8 | 9 |  |
| How severe was your fear of cold? | 0 | 1 | 2 | 3 | 4 | 5 | 6 | 7 | 8 | 9 |  |
| How severe was your sensation of fever? | 0 | 1 | 2 | 3 | 4 | 5 | 6 | 7 | 8 | 9 |  |
| How severe was your spontaneous sweating? | 0 | 1 | 2 | 3 | 4 | 5 | 6 | 7 | 8 | 9 |  |
| How severe was your night sweating? | 0 | 1 | 2 | 3 | 4 | 5 | 6 | 7 | 8 | 9 |  |
| How severe was your dizziness or tinnitus? | 0 | 1 | 2 | 3 | 4 | 5 | 6 | 7 | 8 | 9 |  |
| How severe was your rib-side pain or discomfort? | 0 | 1 | 2 | 3 | 4 | 5 | 6 | 7 | 8 | 9 |  |
| How severe was your belching or acid reflux? | 0 | 1 | 2 | 3 | 4 | 5 | 6 | 7 | 8 | 9 |  |
| How severe was your loss of appetite? | 0 | 1 | 2 | 3 | 4 | 5 | 6 | 7 | 8 | 9 |  |

## Part 2: Do you have the following symptoms? (To be completed by the patient)

We would like to know whether you have experienced the following symptoms in the past week. Please check the appropriate box.

| Do you have any abnormal taste? | None | Bitter | Dry | Sticky |
| --- | --- | --- | --- | --- |
| Do you have any sleep disorders? | Normal | Insomnia | Dream-disturbed sleep | Excessive sleepiness |
| Do you have any urinary abnormalities? (Frequency) | Normal | Oliguria | Polyuria | Nocturia |
| Do you have any urinary abnormalities? (Character) | Normal | Dark and scanty | Clear and long |  |
| Do you have any bowel movement abnormalities? | 1/day | ≥2/day | Every 2 days | ≥1/3 days |
| Do you have any bowel movement abnormalities? (Character) | Soft | Loose | Dry and hard | Inconsistent |
| Do you have any menstrual abnormalities? (for female patients, Flow) | Normal | Light | Heavy |  |
| Do you have any menstrual abnormalities? (Color) | Bright red | Dark red | Deep red |  |
| Do you have any menstrual abnormalities? (Cycle) | Normal | Early | Late |  |

## Part 3: What are your current tongue and pulse conditions? (To be completed by the physician)

Please appropriately record the patient's current tongue and pulse conditions. Check the appropriate box.

| Tongue Coating | Pale red | Pale | Reddish | Purplish-blue |
| --- | --- | --- | --- | --- |
| Tongue Shape | Normal | Tender | Swollen | Thin |
| Tongue Coating Texture | Thin | Thick | Moist | Slippery |
| Tongue Coating Color | White | Yellow | Grey | Black |
| Sub-lingual Vein | Normal | Short and thin | Varicose |  |
| Pulse Condition | Wiry | Slippery | Floating | Deep |

# Attachment 2: Pittsburgh Sleep Quality Index (PSQI)

The following questions pertain to your sleep quality over the past month. Please select the answer that best describes your actual condition during the last month.

1. What time do you usually go to bed at night? ________ (hour).

2. On average, how long does it take you to fall asleep each night? ________ (minutes).

3. What time do you usually wake up in the morning? ________ (hour).

4. What is your actual sleep duration each night? ________ (hours) (Note: This is not equivalent to time spent in bed; decimals are allowed).

|  | Not at all | <1 time/week | 1-2 times/week | ≥ 3 times/week |
| --- | --- | --- | --- | --- |
| A. Difficulty falling asleep (unable to sleep within 30 minutes) |  |  |  |  |
| B. Waking up in the middle of the night or early morning (difficulty returning to sleep) |  |  |  |  |
| C. Getting up to use the bathroom at night |  |  |  |  |
| D. Breathing difficulties |  |  |  |  |
| E. Coughing or snoring loudly |  |  |  |  |
| F. Feeling cold |  |  |  |  |
| G. Feeling hot |  |  |  |  |
| H. Nightmares |  |  |  |  |
| I. Body pain or discomfort |  |  |  |  |
| J. Other factors disturbing sleep (please specify) |  |  |  |  |

Overall, how would you rate your sleep quality over the past month?

1. Very good
2. Fairly good
3. Fairly poor
4. Very poor

How often have you used sleep medications over the past month?

1. Not at all
2. <1 time/week
3. 1-2 times/week
4. ≥ 3 times/week

How often have you felt sleepy during the daytime over the past month?

1. Not at all
2. <1 time/week
3. 1-2 times/week
4. ≥ 3 times/week

How often have you felt a lack of energy to perform tasks during the past month?

1. Never
2. Occasionally
3. Sometimes
4. Often

# Attachment 3: Hospital Anxiety and Depression Scale

1. I feel tense (or distressed).

- Not at all □0
- Occasionally □1
- Most of the time □2
- Almost all the time □3

2. I still take an interest in things that I used to enjoy.

- Not at all □0
- Occasionally □1
- Most of the time □2
- Almost all the time □3

3. I get a sort of frightened feeling as if something awful is about to happen.

- Not at all □0
- Occasionally □1
- Most of the time □2
- Almost all the time □3

4. I can laugh and see the funny side of things.

- Not at all □0
- Occasionally □1
- Most of the time □2
- Almost all the time □3

5. Worrying thoughts go through my mind.

- Not at all □0
- Occasionally □1
- Most of the time □2
- Almost all the time □3

6. I feel cheerful.

- Not at all □0
- Occasionally □1
- Most of the time □2
- Almost all the time □3

7. I can sit at ease and feel relaxed.

- Not at all □0
- Occasionally □1
- Most of the time □2
- Almost all the time □3

8. I feel as if I have slowed down.

- Not at all □0
- Occasionally □1
- Most of the time □2
- Almost all the time □3

9. I get a sort of frightened feeling like "butterflies" in the stomach.

- Not at all □0
- Occasionally □1
- Most of the time □2
- Almost all the time □3

10. I have lost interest in my appearance.

- Not at all □0
- Occasionally □1
- Most of the time □2
- Almost all the time □3

11. I feel restless as I have to be on the move.

- Not at all □0
- Occasionally □1
- Most of the time □2
- Almost all the time □3

12. I look forward with enjoyment to things.

- Not at all □0
- Occasionally □1
- Most of the time □2
- Almost all the time □3

13. I get sudden feelings of panic.

- Not at all □0
- Occasionally □1
- Most of the time □2
- Almost all the time □3

14. I can enjoy a good book or radio or TV program.

- Not at all □0
- Occasionally □1
- Most of the time □2
- Almost all the time □3
